# Monocyte Chemoattractant Protein-1-Deficiency Impairs the Expression of IL-6, IL-1β and G-CSF after Transient Focal Ischemia in Mice

**DOI:** 10.1371/journal.pone.0025863

**Published:** 2011-10-21

**Authors:** Jan-Kolja Strecker, Jens Minnerup, Burkhard Gess, E. Bernd Ringelstein, Wolf-Rüdiger Schäbitz, Matthias Schilling

**Affiliations:** 1 Department of Neurology, University of Münster, Münster, Germany; 2 Department of Neurology, Evangelisches Krankenhaus, Bielefeld, Germany; Julius-Maximilians-Universität Würzburg, Germany

## Abstract

Monocyte chemoattractant protein-1 (MCP-1), a chemokine secreted by neurons and astrocytes following stroke is known to aggravate ischemia-related damage. Previous studies revealed that MCP-1-deficient mice develop smaller infarcts and have an improved neurological outcome, whereas mice overexpressing MCP-1 show worsened brain damage and impaired neurological function. The aim of the present study was to elucidate the molecular background of the enhanced recovery in MCP-1-deficient mice after stroke. For this purpose, we (1) performed expression analyses on crucial post-stroke related inflammatory genes in MCP-1-deficient mice compared to wildtype controls, (2) analyzed a possible impact of MCP-1 on astrocyte activation (3) investigated the cellular origin of respective inflammatory cytokines and (4) analyzed the impact of MCP-1 secretion on the migration of both neutrophil granulocytes and T-cells. Here we report that MCP-1-deficiency leads to a shift towards a less inflammatory state following experimental occlusion of the middle cerebral artery including an impaired induction of interleukin-6, interleukin-1β and granulocyte-colony stimulating factor expression as well as a subsequent diminished influx of hematogenous cells. Additionally, MCP-1-deficient mice developed smaller infarcts 36 hours after experimental stroke. Investigations revealed no differences in transcription of tumor necrosis factor-α and astrogliosis 12 and 36 hours after onset of ischemia. These novel results help to understand post ischemic, inflammatory mechanisms and might give further arguments towards therapeutical interventions by modulation of MCP-1 expression in post stroke inflammation.

## Introduction

Increasing evidence suggests that cytokine mediated post stroke inflammation and subsequent influx/action of hematogenous leukocytes contributes to the development of ischemic brain damage and might, therefore, be a promising target for neuroprotective therapies [1]. Regarding the conclusions of previous studies, clot-lysing treatment of stroke could be more beneficial in combination with neuroprotective and/or pro-regenerative treatments [2]. Cerebral ischemia is a multiphasic event involving acute and prolonged inflammatory processes, which play a pivotal role in disease pathogenesis as well as having a significant impact on neurological outcome [3]. Within the first hours after onset of ischemia affected brain cells produce and secrete proinflammatory cytokines including monocyte chemoattractant protein-1 (MCP-1), interleukin-6 (IL-6), interleukin-1β (IL-1β), tumor necrosis factor-α (TNF-α) and granulocyte-colony stimulating factor (G-CSF) [4], [5]. Monocyte chemoattractant protein-1 (also known as CCL-2) is chemotactic for monocytes, hematogenous macrophages, neutrophil granulocytes, memory T-lymphocytes and natural killer cells [6], [7]. Following cerebral ischemia astrocytic and neuronal MCP-1 secretion within the injured tissue is rapidly induced [8], [9]. Importantly, mice lacking MCP-1 show, along with impaired leukocyte influx and reduced infarct volume, an improved neurological outcome [10], whereas overexpression of MCP-1 leads to exacerbated brain injury and increased migration of inflammatory cells [11]. Since the molecular background of neuroprotection in MCP-1-deficient mice still remains poorly understood, the present study aimed to clarify the influence of MCP-1-signaling on the expression of crucial inflammatory mediators IL-6, IL-1β, TNF-α and G-CSF, reactive astrogliosis, infarct size development and the post ischemic migration of neutrophil granulocytes and T-cells.

## Materials and Methods

All animal studies and procedures have been approved by the local governmental authorities (Landesamt für Natur, Umwelt und Verbraucherschutz, NRW, Germany, AZ 8.87-50.10.36.09) and were conducted in accordance with the European Convention for Animal Care and Ethical Use of Laboratory Animals. The number of animals was kept to a minimum needed.

### Animals

Animals were bred and housed in a pathogen-free environment under standard laboratory conditions with a 12 h/12 h light-dark cycle and were allowed free access to food and water. Fifteen male 16–18 week old C57BL/6J (Charles River, Sulzfeld, Germany) and fifteen male MCP-1-deficient mice (The Jackson Labs, Bar Harbor, Maine, USA) were subjected to experimental cerebral ischemia. Animals were sacrificed 12 and 36 hours following MCAO. Two separate groups of sham-operated mice were used as controls. Generation of MCP-1-knock-out mice has been described before [12]. MCP-1-deficient and wildtype animals were backcrossed at least eight times to obtain a pure C57BL/6J background. Tail-clipped DNA was used for genotyping by PCR according to the genotyping protocols for MCP-1 (Jackson Labs).

### Transient focal cerebral ischemia

Animals weighing 25–30 g were anesthetized and maintained with 1.5% isoflurane in 30% O_2_/70% N_2_O. Body temperature was controlled using a rectal temperature probe and maintained at 37°C±0.5°C by a thermostat-controlled heated blanket. Experimental stroke was induced by occlusion of the middle cerebral artery (MCAO), using a modified intraluminal technique [13]. MCAO was performed with a silicone resin-coated (Xantopren; Haeraeus, Dormagen, Germany) 8-0 nylon monofilament (Ethilon; Ethicon, Norderstedt, Germany) which was introduced into a small incision of the left common carotid artery and advanced distal to the carotid bifurcation for temporary occlusion of the middle cerebral artery (30 min). Regional cerebral blood flow (CBF) was continuously monitored using a laser doppler probe (Periflux 5001; Perimed, Stockholm, Sweden) to verify ischemia and reperfusion. The laser doppler probe was positioned, after an incision was made in the temporal muscle overlapping skin, on the superior portion of the temporal bone (centre of ischemic area). The occlusion was established when the CBF dropped below 20% of the pre-MCAO flow-value. All mice showed appropriate signs for successful operation (disturbance in locomotion and/or flexion of the effected affected limb) and were included in the subsequent experimental procedures.

### Tissue preparation

Both, sham operated mice and animals subjected to MCAO used for subsequent experiments (n = 10 per group) were terminally anesthetized with ketamine/xylazine and perfused intracardially with 0.9% NaCl. Brains were quickly removed, embedded in TissueTek (Sakura Finetek, the Netherlands), frozen on dry ice and stored at −80°C. Mice used for immunohistochemical staining (n = 5 per group) were perfused with 4% paraformaldehyde and brains were postfixed for 3 h in 4% PFA and 10% sucrose overnight (4°C) before embedding and freezing. For both, expression studies and immunohistochemical staining 10 µm thin coronal sections through the infarcted area (approximately bregma 1 mm to 0 mm) were cut using a cryostat (Leica CM 3050, Nussloch, Germany). Specimen used for subsequent gene expression analyses were mounted on polyethylenenaphtalate (PEN) membrane coated slides (PALM MicroLaser Systems, Germany); sections for immunohistochemical analysis were mounted on glass slides (SuperFrost, Langenbrinck, Germany).

### Laser capture microdissection

PEN foil glass slides containing specimen for laser capture microdissection were fixed immediately after sectioning, in 96% ethanol at −20°C for 5 min and air-dried for 1 min. Toluidine blue staining was carried out to identify the ischemic area within the ipsilateral hemisphere. Staining was done with a 0.1% Toluidine blue (Sigma-Aldrich, Munich, Germany)/0.1 M sodium phosphate buffered solution (pH 5.5) for 1 min, followed by dehydration in graded ethanol dilutions (70%, 96%, 100%, 30 s each).

**Figure 1:**
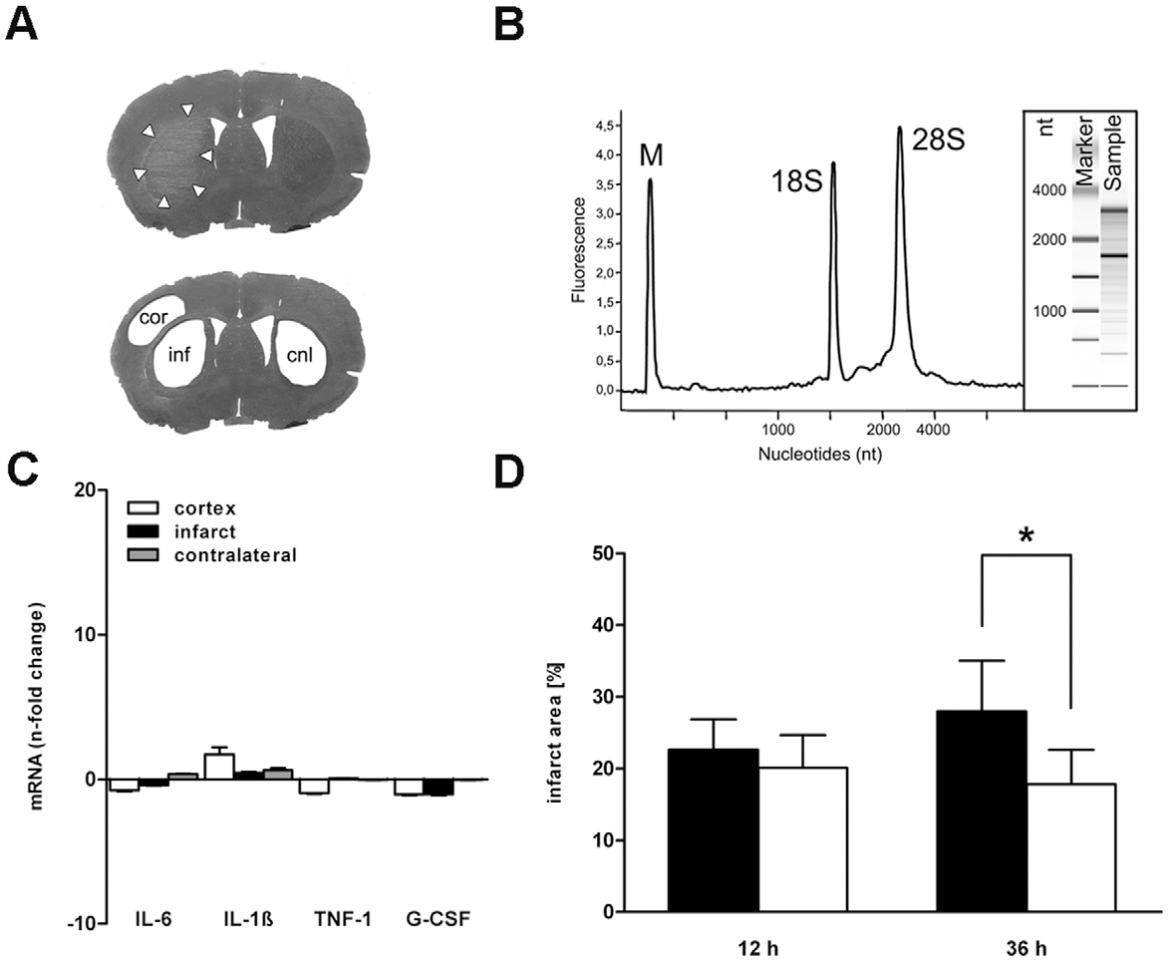
Laser capture microdissection was performed on PEN-membrane mounted and toluidine-stained brain sections. A: Three areas, namely cerebral cortex (cor), infarct core (inf) and contralateral striatum (cnl) were cut out of specimen and total RNA for subsequent analyses was extracted. B: Total RNA integrity of each sample was measured with an Agilent 2100 Bioanalyzer (exemplary data shown). Distinct peaks for 18S and 28S rRNA indicate intact RNA. Only total RNA with a RNA integrity number greater than or equal to 6 was used for subsequent reverse transcription and qRT-PCR. C: Real-Time PCR data showed no significant differences of IL-6, IL-1β, TNF-α and G-CSF expression between wildtype and MCP-1-deficient control animals. D: Infarct size assessed 12 and 36 hours after MCAO. Lesion volume is expressed as percentage of the ipsilateral hemisphere. Values represent mean ± SD, *p<0.05.

Once air-dried, slides were stored at −80°C until further use. Three different areas of interest were isolated using a Zeiss/PALM LMD microscope (Carl Zeiss MicroImaging GmbH, Germany): the ipsilateral cortex (covering the primary motor- and primary somatosensory-cortex), the ipsilateral ischemic area and the contralateral caudate putamen (Fig.1 A). Regions of interest were isolated and collected in microfuge caps containing 45 µl of RNA extraction buffer RLT (Qiagen, Hilden, Germany). To avoid RNA-degradation, lysis buffer was supplemented with 2-mercaptoethanol (143 mM, Sigma-Aldrich, Munich, Germany). Three areas of 20 brain slices from each animal were cut and collected in separate caps for subsequent analysis of gene expression.

### RNA extraction, quality assessement and cDNA-synthesis

Total RNA extraction was carried out using the RNA isolation kit (RNeasy micro kit, Qiagen, Hilden, Germany) following the protocol provided for RNA extraction from microdissected tissue. RNA quality and integrity was measured with the Agilent Bioanalyzer System (Agilent 2100 Bioanalyzer, RNA 6000 PicoLabChip Kit, Agilent Technologies, Santa Clara, CA, USA). RNA samples with a RNA integrity number of six and greater (see Agilent evaluation criterion) were used for cDNA synthesis and subsequent expression analyses (Fig. 1 B). First strand cDNA synthesis was performed using a reverse transcription kit (Quantitect® Reverse Transcription Kit, Qiagen, Hilden, Germany) following the manufacturés procedure.

### Quantitative real-time polymerase chain reaction

All primers were purchased from Qiagen (QuantiTect Assays, Hilden. Germany) and were as follows: glycerinaldehyde-3-phosphate-dehydrogenase (QT01658692); interleukin-1β (QT01048355); tumor necrosis factor-α (QT00104006); interleukin-6 (QT00098875) and granulocyte-colony stimulating factor (QT00105140). To determine mRNA levels of GAPDH, IL-1β, TNF-α, IL-6 and G-CSF, semi-quantitative real-time polymerase chain reactions (qRT-PCR) were performed using SYBR-green fluorescence on ABI PRISM® 7700 RT-PCR-System (Applied Biosystems, Foster City, CA, USA).

### Expression analysis

RT-PCR data were normalized and related for each individual sample to the expression of GAPDH as a housekeeping gene. Relative expression analysis of target genes was carried using the comparative cycle threshold method (ΔΔC_T_). Calculations were performed with the relative expression software tool REST-MCS version 2, which uses the efficiency-corrected comparative crossing point method to estimate a sample expression ratio [14], [15]. Expression changes were reported as a difference relative to the normalized samples of sham-operated animals. In order to reveal possible differences between control animals, an additional comparison of normalized expression patterns between sham-operated MCP-1-deficient and wildtype mice was performed.

### Immunohistochemical staining

Glass mounted coronal sections were postfixed in 4% buffered PFA for 15 min, endogenous peroxidase was blocked by incubation in 3% H_2_O_2_/Methanol for 10 min and nonspecific protein binding was inhibited by incubation with Blocking Reagent (15 min, Roche Diagnostics, Mannheim, Germany). The following antibodies were applied for antigen detection (4°C, overnight): monoclonal antibody directed against microglia and macrophages anti-F4/80, raised in rat (1∶500, Biozol Diagnostica, Eching, Germany), anti-neutrophil antibody 7/4, raised in rat (1∶200, Serotec, Duesseldorf, Germany), monoclonal anti-GFAP antibody directed against astrocytes, raised in mouse (1∶200, Sigma-Aldrich, St. Louis, MO, USA), for detection of neurons monoclonal anti-neuronal nuclei (NeuN), raised in mouse (1∶300, Chemicon Int., Temecula, CA, USA), polyclonal anti-CD3 antibody directed against T-cells, raised in hamster (1∶100, BD Biosciences, Erembodegem, Belgium), polyclonal anti-G-CSF antibody (1∶200, Abcam, Cambridge, UK), polyclonal anti-IL-6 antibody (1∶100, Abcam) and a polyclonal anti-IL-1β antibody (1∶100, Abcam). G-CSF, IL-6 and IL-1β antibodies were raised in rabbit. To detect both anti-neutrophil and anti-F4/80-antibodies we used a biotinylated goat anti-rat antibody (1∶200, Jackson Labs, West Grove, PA, USA) for 45 min at room temperature. For signal amplification of 7/4 and F4/80 signal, brain slices were incubated with horseradish peroxidase/streptavidin (1∶100, DAKO, Glostrup, Denmark) for 45 min and biotinyl tyramide, (1∶100) for 10 min at room temperature. Both microglia and neutrophils were visualized by a streptavidin/fluorescent dye (AlexaFluor488, Molecular Probes, Leiden, the Netherlands). To visualize astrocytes and neurons we used a fluorescent dye-conjugated goat anti-mouse antibody (AlexaFluor488 goat anti-mouse, Molecular Probes). To detect CD-3-antibodies, brain slices were incubated with a biotinylated goat-anti hamster antibody (1∶200, 45 min, room temperature, Jackson Laboratories) and visualized with a streptavidin/fluorescent dye at a dilution of 1∶100 for 45 min at room temperature (AlexaFluor488, Molecular Probes). To detect G-CSF, IL-6 and IL-1β-antibodies a fluorescent dye-conjugated goat-anti rabbit secondary antibody was used at a dilution of 1∶100 for 45 min at room temperature (AlexaFluor594, Molecular Probes). Nuclear counterstaining was done using a fluorescent-preserving mounting medium containing 4′, 6-diamidino-2-phenylindole (DAPI) (Vector, Burlingame, CA, USA). Immunostaining was visualized with a fluorescent microscope (Leica DM microscop, Bensheim, Germany) with appropriate filter sets for AlexaFluor594, AlexaFluor488 and DAPI. Digitizing was done with a Diagnostic SPOT Advanced Camera System (Diagnostic Instr., Sterling Heights, USA). Quantification of IHC-data was carried out by counting cells showing a positive fluorescence signal for IL-6, IL-1β, TNF-α or G-CSF within the questioned areas. Data are presented as percentage of cell numbers in relation to the total number of DAPI-positive cells.

### Infarct size measurement

Comparison of ischemic brain lesion size was carried out using two toluidine stained brain sections of each animal (bregma ∼0.5 mm). Infarct area and the size of the ipsilateral hemisphere were measured using a standard computer assisted analysis technique. Infarct size was calculated as follows: infarcted area/ipsilateral hemisphere*100 and shown as percentage of the ipsilateral hemisphere. Differences between lesioned area sizes of MCP-1-deficient and wildtype animals were evaluated by two-way ANOVA followed by Bonferronis post-hoc test using GraphPad Prism software 5.01 (GraphPad Software, La Jolla, CA, USA). Infarct sizes are presented as mean ± SD and were regarded to be significant if p<0.05.

### Evaluation of reactive astrogliosis

To determine whether MCP-1-deficiency has an influence on astrocyte activation, a separate PFA-fixed coronal section (bregma approximately 0.5 mm) of each animal was stained according to the used protocol for GFAP-immunohistochemistry. GFAP-antibodies were visualized with AlexaFluor594 goat-anti mouse antibodies (Vectashield Labs), nuclear counterstain was done with DAPI. Whole hemisphere images of GFAP-stained sections were taken with a Nikon Eclipse 80i microscope (Nikon GmbH, Duesseldorf, Germany) and the Stereo Investigator Software (MicroBrightField Inc.,Williston, VT, USA). Astrocyte-stained sections were analyzed with respect to differences in immunoreactivity and morphology between wildtype and MCP-1-deficient mice.

### Quantification of migrated neutrophil granulocytes and T-cells

Quantification of migrated neutrophil granulocytes and T-cells was performed by counting and averaging absolute cell numbers within the ipsilateral cortex (Fig.1 A “cor”) of 8 separate brain sections per animal. Differences between neutrophil and T-cell counts were evaluated by the analysis of variance followed by Bonferronis post-hoc test using GraphPad Prism software. Results are presented as mean ± SD and were regarded to be significant if p<0.05.

## Results

### Expression in sham-operated animals

In order to exclude differences of wildtype and MCP-1-deficient constitutional gene expression we compared IL-6, IL-1β, TNF-α and G-CSF mRNA concentration in samples of sham-operated control mice. Data revealed no significant differences of IL-6, IL-1β, TNF-α and G-CSF expression between wildtype and MCP-1-deficient control animals (Fig.1 C).

### Infarct size

Twelve hours following cerebral ischemia no significant difference of infarct size between wildtype (22.63±4.24%) and MCP-1-deficient mice (20.08±4.59%) could be detected (Fig.1 D). In contrast, 36 hours following MCAO, lesion size was significantly increased in wildtype (27.98±7.05%) compared to MCP-1-deficient animals (17.80±4.79%, p<0.05).

### MCP-1-deficiency has no impact on astrocyte activation

**Figure 2:**
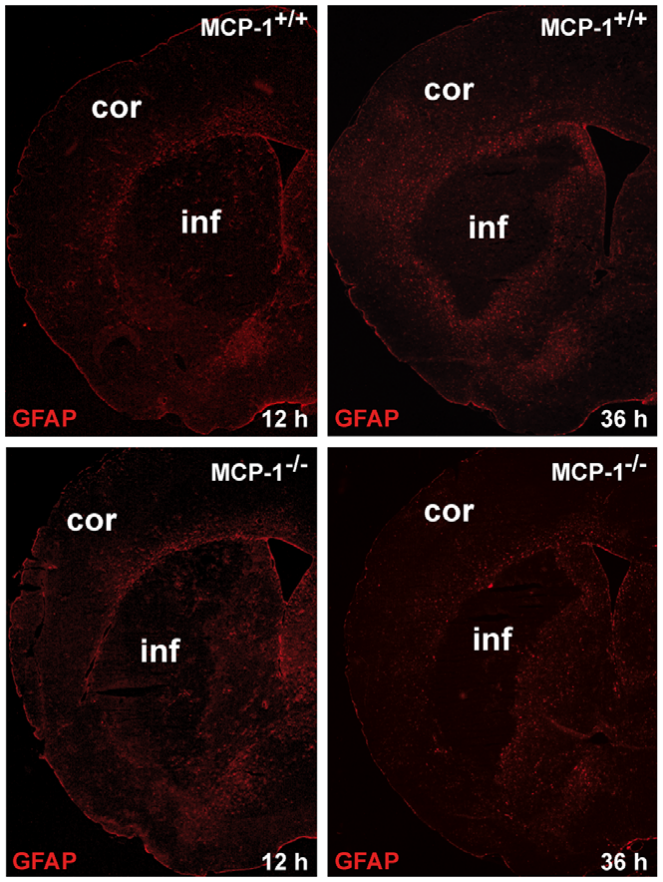
MCP-1-deficiency has no impact on astrocyte activation. Whole hemisphere GFAP-stained images comparing the activation of astrocytes between wildtype and MCP-1-deficient animals. Analyses showed no differences with respect to immunoreactivity and morphology between wildtype and MCP-1-deficient mice. GFAP staining showed the common distribution and activation of astrocytes reacting to oxygen deprivation. Astrogliosis occurred primarily surrounding the infarcted core in both wildtype and MCP-1-deficient mice.

Since astrocytes are known to become activated as a response to a variety of CNS pathologies [16] we investigated a possible influence of MCP-1 on the reactive astrogliosis 12 and 36 hours after cerebral ischemia. Comparing analyses of GFAP-stained coronal sections showed no differences with respect to immunoreactivity and morphology between both investigated groups and time-points. Activated astrocytes showed the common distribution and activation primarily surrounding the infarcted core in both wildtype and MCP-1-deficient mice (Fig.2).

### MCP-1-deficiency impairs IL-6 expression

**Figure 3:**
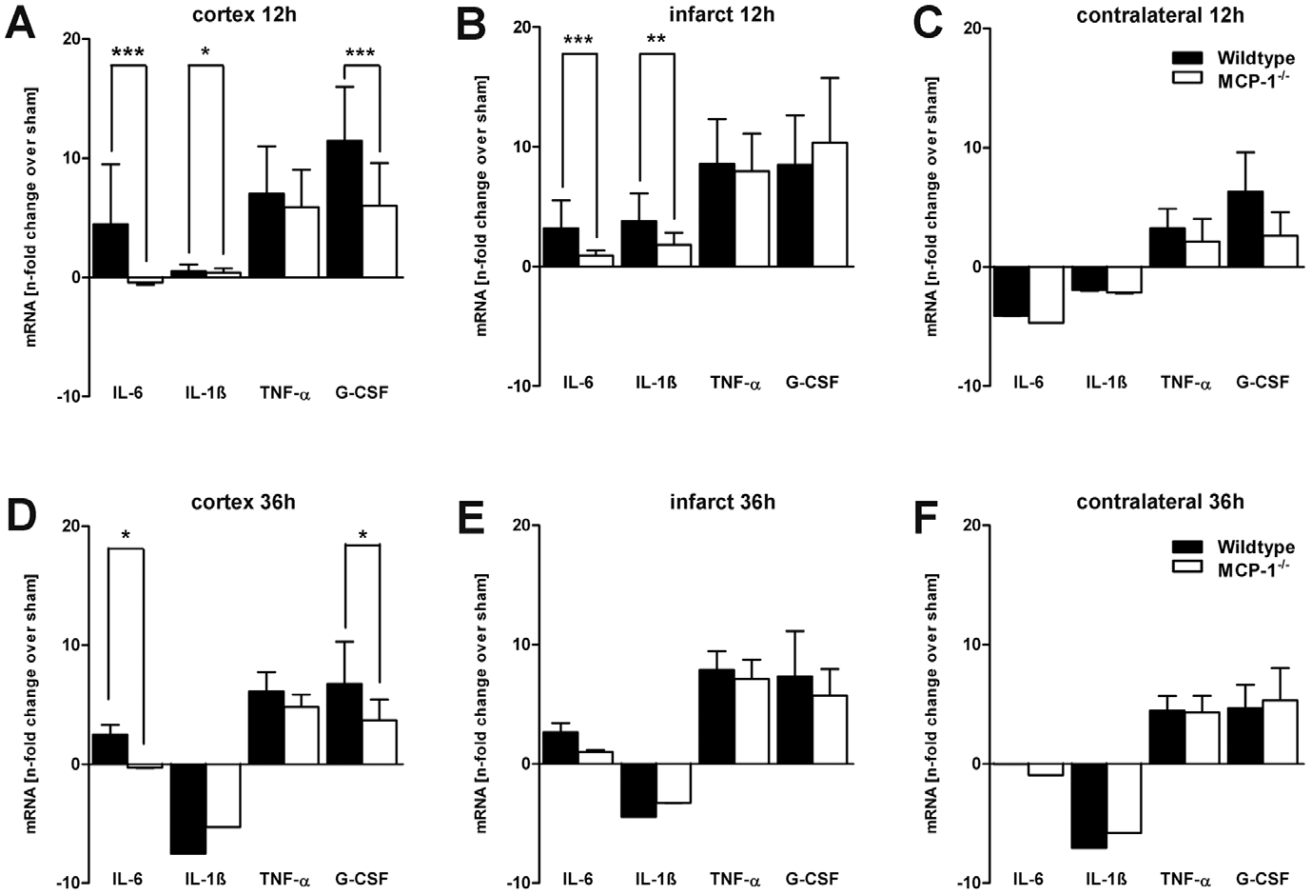
IL-6, IF-1β, TNF-α and G-CSF mRNA expression within three defined microdissected areas. RT-PCR was performed to detect and compare mRNA levels of wildtype and MCP-1-deficient mice within the cerebral cortex, infarcted core and contralateral striatum 12 hours (A−C) and 36 hours (D−F) after focal transient ischemia. Data represent mean levels ± SD of mRNA expression in wildtype and knock-out mice compared to respective sham-operated mice. RT-PCR was repeated twice for each sample. *p<0.05; **p<0.01; ***p<0.001: statistically significant differences between wildtype versus knock-out mice (ANOVA with Bonferronis post hoc-test).

**Figure 4:**
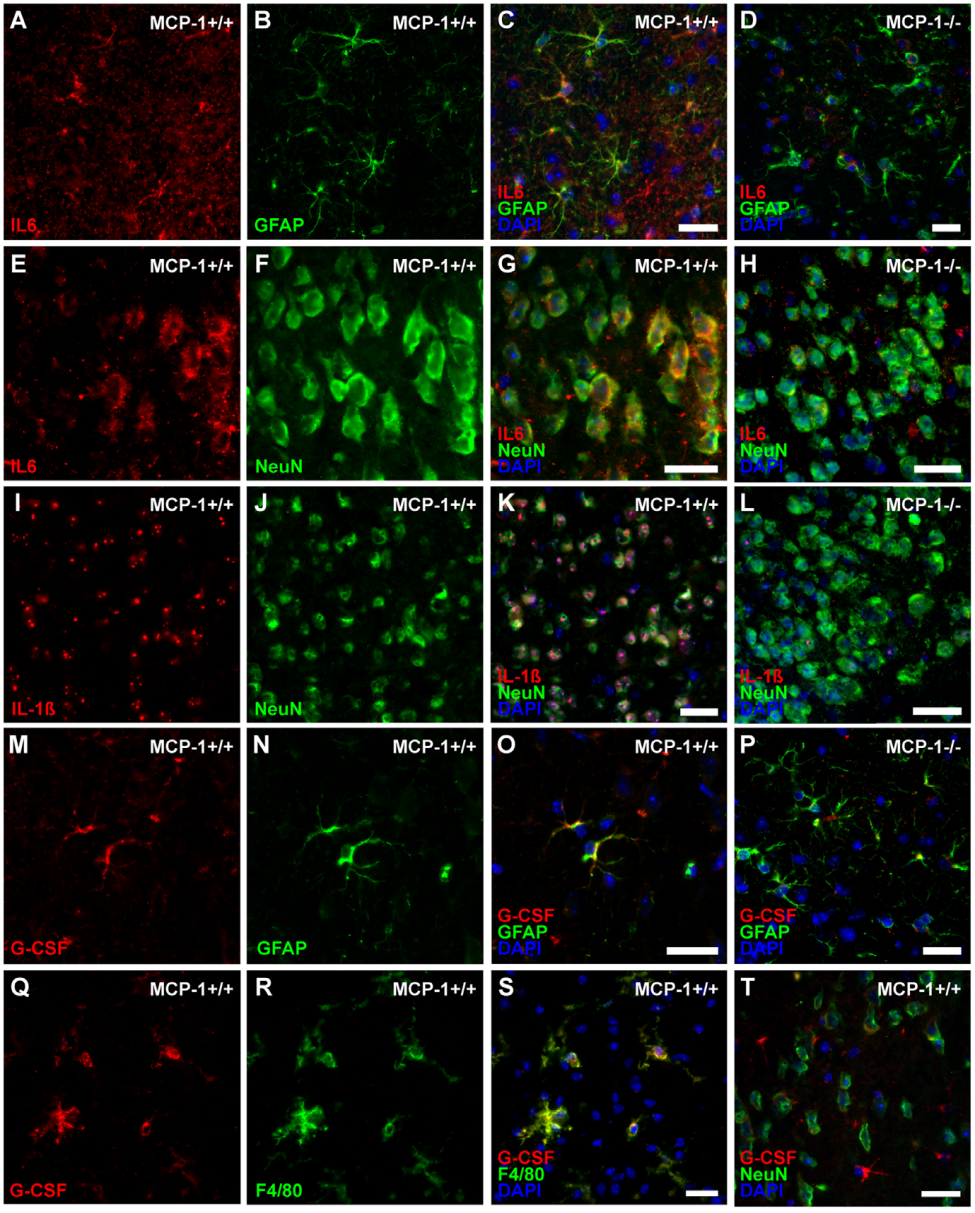
Immunofluorescence-staining within the ipsilateral cerebral cortex revealed astrocytes (A−C) and neurons (E−G) as the main source of interleukin-6-synthesis. In MCP-1-deficient mice, IL-6 signal pattern also showed double positive signals colocalized with astroctyes (D) and neurons (H) but with reduced intensity indicating a diminished IL-6 concentration on the translational level. Expression of IL-1β within the ischemic core was mainly restricted to neurons (I−K) in both wildtype and MCP-1-deficient mice, the latter displaying a reduced IL-1β-fluorescence intensity confirming the results of the expression analyses (L). Shrinked cell morphology indicates apoptotic processes within the infarcted core. Double immunofluorescence-staining also showed co-localization of G-CSF-positive cells and astrocytes (M−O), microglia (Q−S) and occasionally, neurons (T). In MCP-1-deficient mice astrocytic G-CSF secretion was clearly diminished, visualized by the reduction of fluorescence signal intensity reveals (P). Scale bar = 25 µm.

**Figure 5:**
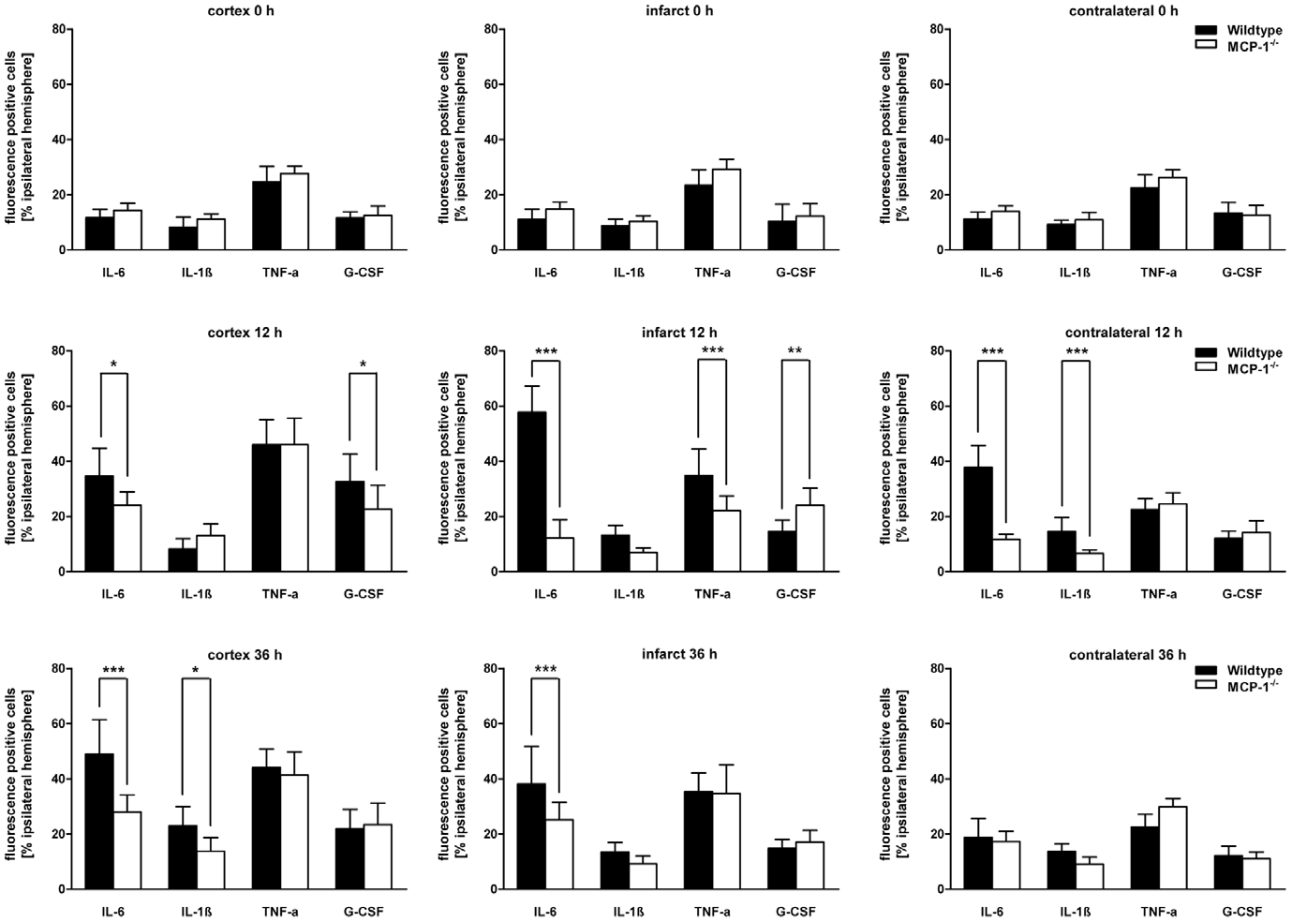
Quantification of IL-6, IF-1β, TNF-α and G-CSF expressing cells. IL-6, IF-1β, TNF-α and G-CSF-positive cells as well as DAPI-positive cells were counted 0, 12 and 36 hours following MCAO. Cell numbers are presented as percentage in relation to the total DAPI-positive cell number within the respective area. Data are shown as mean±SD. *p<0.05; **p<0.01; ***p<0.001: statistically significant differences between wildtype versus MCP-1-deficient animals (ANOVA followed by Bonferronis post hoc-test).

To investigate whether MCP-1 expression is associated with an induction of IL-6, we measured and compared IL-6-mRNA concentrations within three different brain areas of wildtype and MCP-1-deficient mice. Twelve hours after onset of MCAO a considerable induction of IL-6 was found within the cortex and infarct core of wildtype animals. In contrast, MCP-1-deficient animals showed a severely impaired IL-6 expression in both ipsilateral cortex (p<0.01) and infarcted core (p<0.01, Fig.3 A, B, C). Thirty-six hours after MCAO IL-6 expression patterns showed no significant change compared to the earlier time-point in both wildtype and MCP-1-deficient animals. IL-6 expression remained elevated in both wildtype cerebral cortex (p<0.05) and infarcted core, whereas samples of MCP-1-deficient mice showed almost no IL-6 expression in all investigated areas (Fig.3 D, E, F-). Next, we performed dual immunohistochemical analysis of IL-6 and cell markers for neurons, astrocytes, microglia/macrophages and neutrophil granulocytes. Double fluorescence analysis revealed colocalizations of IL-6 with GFAP and NeuN within the ipsilateral cerebral cortex and infarcted core indicating that IL-6 is particularly expressed by astrocytes and neurons after cerebral ischemia (Fig.4 A, B, C, D, E, F, G, H). In concordance with the results of mRNA expression we found significantly reduced cell numbers positive for IL-6 in MCP-1 deficient mice within the ipsilateral cortex and the ischemic core at both investigated time-points (Fig.5). Regarding the contralateral hemisphere, IL-6 positive cells increased in wildtype animals 12 hours after MCAO, whereas cell counts remained on the level of the control group in MCP-1-deficient mice at both time-points.

### MCP-1-deficiency leads to an impaired expression of IL-1ß

To investigate whether MCP-1 signalling determines post-ischemic IL-1β regulation we compared relevant mRNA levels within the three defined areas 12 and 36 hours after MCAO. In wildtype mice, IL-1β was expressed on a significantly higher level within the cerebral cortex (p<0.05) and the ischemic core (p<0.01) 12 hours after MCAO (Fig.3 A, B). Data indicates that IL-1β induction is mainly restricted to the infarcted core as mRNA-concentrations within the cortex and the contralateral hemisphere were at a low level or even downregulated. In contrast, 36 hours following MCAO IL-1β expression was severely downregulated in both MCP-1-deficient and wildtype animals and all investigated areas (Fig.3 D, E, F). However, no significant differences between the two groups could be observed. Immunohistochemical double staining revealed neurons as main source of IL-1β (Fig.4 I, J, K, L). In wildtype mice, occasional colocalization of IL-1β and astrocytic marker GFAP could be detected but was entirely missing in MCP-1-deficient animals (not shown). In concordance, IL-1β fluorescence signal intensity was reduced in MCP-1-deficient animals compared to the wildtype group (Fig.4 L), although IL-1β cell quantification showed no differences between the investigated groups (Fig.5). Furthermore, in wildtype animals no significant changes compared to the respective control group could be observed except increased numbers of IL-1β expressing cells within the contralateral hemisphere at 12 hours (p<0.001) and the perilesional cortex 36 hours (p<0.05) following MCAO.

### TNF-α expression is not affected by MCP-1-deficiency

As expected, TNF-α expression was upregulated following cerebral ischemia. Admittedly, 12 and 36 hours after transient cerebral ischemia TNF-α showed no significant differences between wildtype mice and MCP-1-deficient animals (Fig.3 A, B, C). Thirty-six hours after cerebral ischemia, TNF-α mRNA remained elevated in both investigated groups and showed no differences in comparison to the earlier time point. Immunohistochemistry revealed neurons as the main source of TNF-α. Quantitative analysis confirmed transcriptional data as no difference in cell number could be observed except within the infarcted core, which showed less TNF-α positive cells in MCP-1-deficient animals 12 hours after MCAO (p<0.001, Fig.5).

### G-CSF expression is affected by MCP-1

Following cerebral ischemia, upregulation of granulocyte colony-stimulating factor has been reported in both rodents and humans [17], [18]. Therefore, we examined a possible influence of MCP-1 signalling on the expression of G-CSF. Indeed, 12 and 36 hours after transient MCAO, G-CSF was induced and upregulated in both wildtype and MCP-1-deficient mice (Fig.3). Analysis within the ipsilateral cortex revealed a reduced induction of G-CSF in MCP-1-deficient mice compared to the wildtype group 12 hours (p<0.001) after MCAO. No significant difference in G-CSF expression could be detected within the infarct core at both investigated time-points. Furthermore, G-CSF was upregulated within the contralateral hemispheres of both wildtype and MCP-1-deficient mice. Thirty-six hours after onset of cerebral ischemia G-CSF mRNA-concentration declined within the ipsilateral hemisphere. However, expression patterns showed similar characteristics compared to the earlier time-point showing an elevated G-CSF expression in wildtype mice within the cerebral cortex (p<0.05, Fig.3 D). Dual immunohistochemical staining revealed astrocytes and both ramified and activated microglia as the main source of G-CSF (Fig.4 M, N, O, P, Q, R, S, T). Quantitative analysis showed few G-CSF-positive stained cells in sham-operated mice (Fig.5). Twelve hours following MCAO, confirming transcriptional data, cell numbers within the perilesional cortex increased in both analyzed groups and declined 36 hours after cerebral ischemia. Quantification within the infarcted core showed no significant increase of G-CSF positive cells compared to the sham-operated group except in MCP-1-deficient mice 12 hours after onset of the occlusion (p<0.01). Furthermore, no quantitative changes of G-CSF-positive cells could be detected within the contralateral hemisphere in both groups and time-points.

### MCP-1-deficiency leads to a reduced influx of neutrophil granulocytes and T-cells

**Figure 6:**
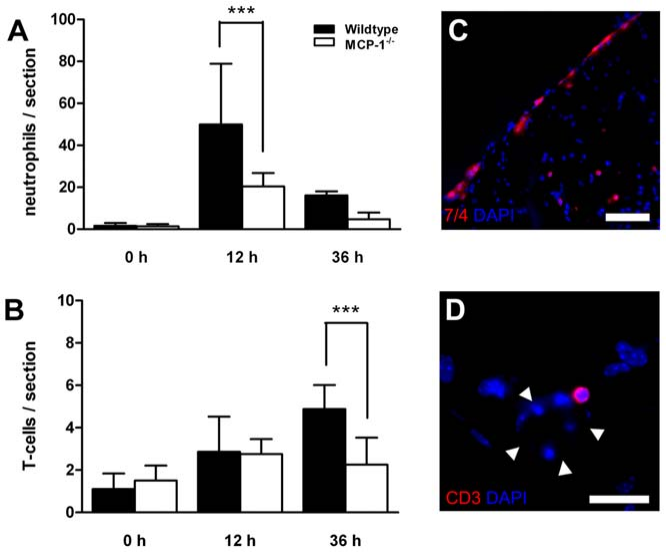
MCP-1-deficiency leads to an impaired influx of neutrophil granulocytes and T-cells. (A) Quantitative assessment of migrated neutrophils within ipsilateral cortex (*cor*, fig.1 A) following 30 min transient middle cerebral artery occlusion. Values are obtained counting the absolute number of cortical 7/4-positive cells in 8 sections per animal. Twelve hours after MCAO, MCP-1-deficient mice showed an impaired influx of neutrophil granulocytes within the ipsilateral cortex compared to wildtype controls (*p<0.001). (B) Quantitative analysis of T-cell migration within the ipsilateral cortex of MCP-1-deficient and wildtype mice. Thirty-six hours after MCAO T-cell numbers were significantly lower in MCP-1-deficient animals. (C) Immunohistochemistry of neutrophil granulocytes within the ipsilateral cortex. Representative 7/4 stained image taken from a wildtype animal. Following transient cerebral ischemia most neutrophil granulocytes were found within the meningeal layers and the cortical tissue. Scale bar = 100 µm. (D) T-cell within the ipsilateral cortex of a wildtype mouse. Arrowheads mark the border of a blood vessel. Nuclear counterstain with DAPI (blue). Scale bar = 25 µm.

As a result of oxygen deprivation and subsequent expression of chemokines and cytokines, various hematogenous cells are attracted into the ischemic tissue and influence the subsequent inflammatory response. In this study, we analyzed whether the altered expression of IL-6, IL-1β and G-CSF in MCP-1-deficient mice has an influence on the post-stroke migration of neutrophil granulocytes and T-cells. For this purpose, we counted and compared the numbers of migrated neutrophil granulocytes and T-cells within the ipsilateral cortex of wildtype and MCP-1-deficient mice. We detected an accumulation of 7/4-positive cells within the ipsilateral cortex and the meningeal layers in both examined groups of animals (Fig.6 C). Cell number analysis revealed a reduced influx of neutrophil granulocytes in MCP-1-deficient mice 12 hours following MCAO (p<0.001, Fig.6 A). Twelve hours after cerebral ischemia few T-cells could be seen in both wildtype and MCP-1-deficient mice. The majority of detected T-cells seemed to be in close vicinity to blood vessels or attached to the respective endothelium (Fig.6 D). Quantitative analyses revealed a significant difference 36 hours after MCAO as T-cell numbers increased in wildtype but not MCP-1-deficient mice (p<0.01).

## Discussion

The mechanisms by which MCP-1 contributes to final infarct size and neurological outcome after stroke remain largely unknown. The present study investigated the influence of MCP-1 signalling on the expression profile of stroke relevant inflammatory cytokines, astrocytosis, lesion development and its effects on the migration of neutrophil granulocytes and T-cells. As development of infarct volume is completed 24 hours following cerebral ischemia [19], we decided to investigate the expression and secretion pattern of the questioned proteins 12 and 36 hours after MCAO. Here, we report that MCP-1-deficiency impairs the induction of IL-6, IL-1β and partially G-CSF early after cerebral ischemia, resulting in a reduced influx of neutrophil granulocytes and T-cells. Interestingly, we observed the altered inflammatory response in MCP-1-deficient mice, particularly 12 hours after cerebral ischemia, as the transcription rate of most of the investigated genes declined and equalized between wildtype and MCP-1-deficient mice 36 hours after MCAO. Importantly, a lack of MCP-1 did not result in an impaired astrogliosis or varying infarct size 12 hours following MCAO, concluding that diminished transcription of IL-6, IL-1β and G-CSF is not caused by delayed astrocyte activation or smaller lesion size. In a previous study, we found no differences in the infarct volume between wildtype and MCP-1-mice during the early phase of lesion development [9]. However, confirming the findings of the present study, MCP-1-deficient mice showed significant smaller infarct volumes 48 hours after MCAO. The role of endogenous interleukin-6 in post-stroke inflammation and development of brain tissue damage still remains unclear. Mice lacking IL-6 showed no difference in both infarct size and neurological function 24 hours following MCAO [20], whereas clinical studies report a worsening influence of elevated serum IL-6 in stroke patients [21], [22]. Findings of another study even showed an anti-apoptotic and neuroprotective effect of endogenous IL-6 on oxygen deprivated neurons following MCAO [23]. Our findings reveal an extensive breakdown of IL-6-transcription in MCP-1-deficient mice. This could be explained by results of previous studies, which showed that activation of the interleukin-6 receptor IL-6R induces MCP-1 expression and further secretion of IL-6. This creates an autocrine stimulation loop via the JAK/STAT signal transduction pathway for both IL-6 and MCP-1, the latter resulting in an autocrine induction via its receptor CCR-2 [24], [25]. Our results indicate that a lack of MCP-1 constrains these loops, resulting in a reduced expression of IL-6 in neurons and astrocytes within the ipsilateral cortex and ischemic striatum.

Interleukin-1β has been shown to negatively contribute to ischemic, excitotoxic and traumatic brain injury. Intraventricular injection of IL-1β excacerbates brain damage following occlusion of the middle cerebral artery [4], while immuno-neutralization of IL-1β leads to smaller infarcts [26]. Our results show that lack of MCP-1 impairs IL-1β expression especially within the ischemic core 12 hours after onset of cerebral ischemia, which is in agreement with findings of previous studies demonstrating a causal relationship between diminished IL-1β and smaller infarct sizes [27], [28]. Furthermore, a recent study reported a direct link between activation of JAK/STAT pathway and subsequent induction of IL-1β and IL-6 in murine macrophages. In accordance with our findings, induction of latter signal transduction pathway led to an elevated IL-1β and IL-6 production but failed to induce TNF-α [29].

In contrast, G-CSF is known to suppress various elements of the post stroke inflammatory response including downregulation of interleukin-1β and reduction of edema formation [30], [31]. Several other studies showed further beneficial effects of G-CSF-treatment following experimental cerebral ischemia such as reduced infarct volume, improved functional outcome, neurogenesis and anti-apoptotic effects [32], [17], [18]. Surprisingly, we found that G-CSF transcription is downregulated in MCP-1-deficient mice within the ipsilateral cortex. However, these results are in accordance with our findings of a diminished influx of neutrophil granulocytes within the ipsilateral cortex of MCP-1-deficient mice as neutrophil proliferation and migration is mainly driven by G-CSF [33]. Following cerebral ischemia, neutrophil migration has been shown to be a critical factor in subsequent damage development. Several studies report deleterious contribution concerning neutrophil action in brain damage pathology for instance by secreting matrix metallo-proteinases which degrade extracellular matrix and blood-brain-barrier (BBB) proteins, resulting in disruption of BBB integrity, aggravation of edema formation, hemorrhagic transformation and inflammation [34], [35], [36]. Numerous publications describe a relationship between the number of attracted neutrophils, final infarct volume and neurological outcome in which less neutrophils denoted a milder course of disease [37], [38]. This is in accordance with our findings and previous reports [6] revealing an impaired influx of neutrophil granulocytes in MCP-1-deficient mice.

Since T-lymphocyte action has been reported to aggravate post ischemia lesion size [39], we also investigated a possible influence of MCP-1 on the migration of T-cells. Indeed, mice lacking MCP-1 showed an impaired influx of T-cells 36 hours after cerebral ischemia. As TNF-α is an important factor for T-lymphocyte activation and response [40] the diminished influx of T-cells in MCP-1-deficient mice could be explained by the reduced cell number expressing TNF-α within the infarcted core 12 hours after MCAO. However, we did not found an altered expression profile of TNF-α comparing the investigated groups. There is evidence that JAK/STAT signalling directly induces MCP-1 expression via binding of STAT to the MCP-1 promotor [25]. Since expression and induction of IL-6, IF-1β and G-CSF are also connected to the JAK/STAT signal transduction pathway, shifting towards a less inflammatory state in MCP-1-deficient mice could be due to an interruption of JAK/STAT-dependent autocrine loops. Especially during the early post ischemic phase, this might result in smaller infarct volumes, reduced loss of BBB integrity, less influx of hematogenous cells and improved neurological outcome in MCP-1-deficient mice.

Although it is not possible to clarify all mechanisms associated with smaller infarcts, neuroprotective effects and improved clinical outcome in MCP-1-deficient mice, we can conclude that the induction of inflammation-related cytokines such as IL-6, IF-1β and G-CSF is impaired in MCP-1-deficient mice during the early inflammatory phase, resulting in a decreased migration of hematogenous cells and reduced lesion size.

## References

[1] Lin N, Friedlander RM (2009). T-cell activation in ischemic stroke: a new therapeutic target for delayed infarct expansion?. Neurosurgery.

[2] Kriz J, Lalancette-Hébert M (2009). Inflammation, plasticity and real-time imaging after cerebral ischemia.. Acta Neuropathol.

[3] Gelderblom M, Leypoldt F, Steinbach K, Behrens D, Choe CU (2009). Temporal and spatial dynamics of cerebral immune cell accumulation in stroke.. Stroke.

[4] Touzani O, Boutin H, LeFeuvre R, Parker L, Miller A (2002). Interleukin-1 influences ischemic brain damage in the mouse independently of the interleukin-1 type I receptor.. J Neurosci.

[5] Schneider A, Krüger C, Steigleder T, Weber D, Pitzer C (2005). The hematopoietic factor G-CSF is a neuronal ligand that counteracts programmed cell death and drives neurogenesis.. J Clin Invest.

[6] Schilling M, Strecker JK, Schäbitz WR, Ringelstein EB, Kiefer R (2009). Effects of monocyte chemoattractant protein 1 on blood-borne cell recruitment after transient focal cerebral ischemia in mice.. Neuroscience.

[7] Haile WB, Echeverry R, Wu J, Yepes M (2010). The interaction between tumor necrosis factor-like weak inducer of apoptosis and its receptor fibroblast growth factor-inducible 14 promotes the recruitment of neutrophils into the ischemic brain.. J Cereb Blood Flow Metab.

[8] Che X, Ye W, Panga L, Wu DC, Yang GY (2001). Monocyte chemoattractant protein-1 expressed in neurons and astrocytes during focal ischemia in mice.. Brain Res.

[9] Deng YY, Lu J, Ling EA, Kaur C (2009). Monocyte chemoattractant protein-1 (MCP-1) produced via NF-kappaB signaling pathway mediates migration of amoeboid microglia in the periventricular white matter in hypoxic neonatal rats.. Glia.

[10] Hughes PM, Allegrini PR, Rudin M, Perry VH, Mir AK (2002). Monocyte chemoattractant protein-1 deficiency is protective in a murine stroke model.. J Cereb Blood Flow Metab.

[11] Chen Y, Hallenbeck JM, Ruetzler C (2003). Overexpression of monocyte chemoattractant protein 1 in the brain exacerbates ischemic brain injury and is associated with recruitment of inflammatory cells.. J Cereb Blood Flow Metab.

[12] Lu B, Rutledge BJ, Gu L, Fiorillo J, Lukacs NW (1998). Abnormalities in monocyte recruitment and cytokine expression in monocyte chemoattractant protein 1-deficient mice.. J Exp Med.

[13] Hata R, Mies G, Wiessner C (1998). A reproducible model of middle cerebral artery occlusion in mice: hemodynamic, biochemical, and magnetic resonance imaging.. J Cereb Blood Flow Metab.

[14] Pfaffl MW (2001). A new mathematical model for relative quantification in real-time RT-PCR.. Nucleic Acids Res.

[15] Pfaffl MW, Horgan GW, Dempfle L (2002). Relative expression software tool (REST) for group-wise comparison and statistical analysis of relative expression results in real-time PCR.. Nucleic Acids Res.

[16] Pekny M, Nilsson M (2005). Astrocyte activation and reactive gliosis.. Glia.

[17] Minnerup J, Sevimli S, Schäbitz WR (2009). Granulocyte-colony stimulating factor for stroke treatment: mechanisms of action and efficacy in preclinical studies.. Exp Transl Stroke Med.

[18] Schneider A, Krüger C, Steigleder T, Weber D, Pitzer C (2005). The hematopoietic factor G-CSF is a neuronal ligand that counteracts programmed cell death and drives neurogenesis.. J Clin Invest.

[19] Hata R, Maeda K, Hermann D, Mies G, Hossmann KA (2000). Dynamics of regional brain metabolism and gene expression after middle cerebral artery occlusion in mice.. J Cereb Blood Flow Metab.

[20] Clark WM, Rinker LG, Lessov NS, Hazel K, Hill JK (2000). Lack of interleukin-6 expression is not protective against focal central nervous system ischemia.. Stroke.

[21] Acalovschi D, Wiest T, Hartmann M, Farahmi M, Mansmann U (2003). Multiple levels of regulation of the interleukin-6 system in stroke.. Stroke.

[22] Montaner J, Rovira A, Molina CA, Arenillas JF, Ribó M (2003). Plasmatic level of neuroinflammatory markers predict the extent of diffusion-weighted image lesions in hyperacute stroke.. J Cereb Blood Flow Metab.

[23] Yamashita T, Sawamoto K, Suzuki S, Suzuki N, Adachi K (2005). Blockade of interleukin-6 signaling aggravates ischemic cerebral damage in mice: possible involvement of Stat3 activation in the protection of neurons.. J Neurochem.

[24] Watanabe S, Mu W, Kahn A, Jing N, Li JH (2004). Role of JAK/STAT pathway in IL-6-induced activation of vascular smooth muscle cells.. Am J Nephrol.

[25] Potula HS, Wang D, Quyen DV, Nikhlesh KS, Venkatesh KS (2009). Src-dependent STAT-3-mediated expression of monocyte chemoattractant protein-1 is required for 15(S)-hydroxyeicosatetraenoic acid-induced vascular smooth muscle cell migration.. J Biol Chem.

[26] Loddick SA, Rothwell NJ (1996). Neuroprotective effects of human recombinant interleukin-1 receptor antagonist in focal cerebral ischaemia in the rat.. J Cereb Blood Flow Metab.

[27] Bowen KK, Naylor M, Vemuganti R (2006). Prevention of inflammation is a mechanism of preconditioning-induced neuroprotection against focal cerebral ischemia.. Neurochem Int.

[28] Shin JA, Park EM, Choi JS, Seo SM, Kang JL (2009). Ischemic preconditioning-induced neuroprotection is associated with differential expression of IL-1beta and IL-1 receptor antagonist in the ischemic cortex.. J Neuroimmunol.

[29] Samavati L, Rastogi R, Du W, Hüttemann M, Fite A (2009). STAT3 tyrosine phosphorylation is critical for interleukin 1 beta and interleukin-6 production in response to lipopolysaccharide and live bacteria.. Mol Immunol.

[30] Gibson CL, Jones NC, Prior MJ, Bath PM, Murphy SP (2005a). G-CSF suppresses edema formation and reduces interleukin-1beta expression after cerebral ischemia in mice.. J Neuropathol Exp Neurol.

[31] Gibson CL, Bath PM, Murphy SP (2010). G-CSF administration is neuroprotective following transient cerebral ischemia even in the absence of a functional NOS-2 gene.. J Cereb Blood Flow Metab.

[32] Gibson CL, Bath PM, Murphy SP (2005b). G-CSF reduces infarct volume and improves functional outcome after transient focal cerebral ischemia in mice.. J Cereb Blood Flow Metab.

[33] Lieschke GJ, Grail D, Hodgson G, Metcalf D, Stanley E (1994). Mice lacking granulocyte colony-stimulating factor have chronic neutropenia, granulocyte and macrophage progenitor cell deficiency, and impaired neutrophil mobilization.. Blood.

[34] McColl BW, Rothwell NJ, Allan SM (2007). Systemic inflammatory stimulus potentiates the acute phase and CXC chemokine responses to experimental stroke and exacerbates brain damage via interleukin-1- and neutrophil-dependent mechanisms.. J Neurosci.

[35] Sevimli S, Diederich K, Strecker JK, Schilling M, Klocke R (2009). Endogenous brain protection by granulocyte-colony stimulating factor after ischemic stroke.. Exp Neurol.

[36] Murikinati S, Jüttler E, Keinert T, Ridder DA, Muhammad S (2010). Activation of cannabinoid 2 receptors protects against cerebral ischemia by inhibiting neutrophil recruitment.. FASEB J.

[37] Buck BH, Liebeskind DS, Saver JL, Bang OY, Yun SW (2008). Early neutrophilia is associated with volume of ischemic tissue in acute stroke.. Stroke.

[38] Gautier S, Ouk T, Petrault O, Caron J, Bordet R (2009). Neutrophils contribute to intracerebral haemorrhages after treatment with recombinant tissue plasminogen activator following cerebral ischaemia.. Br J Pharmacol.

[39] Yilmaz G, Arumugam TV, Stokes KY, Granger DN (2006). Role of T lymphocytes and interferon-gamma in ischemic stroke.. Circulation.

[40] Gee JM, Kalil A, Shea C, Becker KJ (2007). Lymphocytes: potential mediators of postischemic injury and neuroprotection.. Stroke.

